# Bidirectional crosstalk between ER stress and lipid metabolism: From proteostasis to tumor adaptation

**DOI:** 10.1038/s41420-025-02878-y

**Published:** 2025-12-05

**Authors:** Yueling Wu, Huijuan Luo, Zhiwei Pan, Weiping Chen, Lei Bi

**Affiliations:** 1 Faculty of Chinese Medicine, Macau University of Science and Technology, Macau, 999078 China; 2 School of Chinese Medicine, Nanjing University of Chinese Medicine, Nanjing, 210023 China; 3 Shanxi Key Laboratory of Chinese Medicine Encephalopathy, Shanxi University of Chinese Medicine, Jinzhong, 030619 China

**Keywords:** Cancer metabolism, Membrane lipids, Endoplasmic reticulum

## Abstract

Endoplasmic reticulum (ER) stress is a central adaptive response that maintains proteostasis under diverse metabolic and environmental challenges. In cancer, ER stress and lipid metabolism form a tightly coupled, bidirectional regulatory network that integrates protein quality control with lipid remodeling. Through the unfolded protein response (UPR), ER stress reprograms lipid synthesis, oxidation, and storage to sustain energy balance and membrane integrity. Conversely, dysregulated lipid accumulation disrupts ER homeostasis and amplifies stress signaling, creating a feedback loop between metabolic and proteostatic imbalance. Proteostasis systems, including the ubiquitin-proteasome system (UPS) and autophagy, cooperate with UPR signaling to fine-tune this adaptive balance and enhance tumor survival under stress. This review highlights the bidirectional crosstalk between ER stress and lipid metabolism from the perspective of proteostasis-driven tumor adaptation and summarizes emerging therapeutic strategies such as small-molecule modulators, natural products, and combination therapies that target this adaptive network to overcome drug resistance and improve cancer treatment.

## Facts

- ER stress and lipid metabolism are bidirectionally coupled in cancer cells, forming a dynamic regulatory loop that maintains proteostasis, supports metabolic adaptation, and contributes to therapeutic resistance.
- The UPR—via PERK, IRE1α, and ATF6—reprograms lipid metabolism by modulating fatty acid synthesis and oxidation, cholesterol homeostasis, and lipid droplet dynamics.
- Dysregulated lipid accumulation, including saturated fatty acids and cholesterol overload, disrupts ER membrane integrity and calcium homeostasis, aggravating ER stress and reinforcing UPR signaling.
- Targeting the ER stress-lipid metabolism axis through small-molecule inhibitors, natural products, or combination therapies represents a promising approach to restrain tumor adaptation and overcome drug resistance.

## Open questions

- Do the three UPR branches exhibit compensatory or specialized roles in regulating lipid metabolism during tumor adaptation? Does the order or dominance of their activation vary with the type or intensity of ER stress?
- Under different tumor metabolic conditions, what key factors determine the strength and outcome of the “lipotoxicity–ER stress” feedback loop—favoring either adaptation or apoptosis?
- The cooperative role of ER stress and lipid metabolism in promoting tumor adaptation and therapy resistance remains incompletely understood. How can co-targeting strategies be designed to selectively impair tumor survival without inducing excessive stress in normal cells?
- Can natural compounds serve as multitarget modulators of the ER stress-lipid metabolism axis? Further studies are needed to optimize their pharmacological properties and enhance selectivity.

## Introduction

Tumor cells experience substantial proteostatic and metabolic stress from rapid proliferation, high protein synthesis, and intensified membrane biogenesis under nutrient- and oxygen-deprived conditions [1]. These challenges often disrupt endoplasmic reticulum (ER) function, leading to sustained ER stress (ERS), a recognized hallmark of cancer [2]. To maintain cellular homeostasis, cancer cells activate adaptive stress responses, including the unfolded protein response (UPR), the ubiquitin–proteasome system (UPS), and autophagy. Together, these pathways form an integrated proteostasis network that coordinates protein folding, degradation, and recycling with cellular metabolism [3].

Accumulating evidence highlights the bidirectional crosstalk between ER stress signaling and lipid metabolic remodeling. The UPR not only regulates protein quality control but also modulates lipid biosynthesis, fatty acid oxidation, and lipid droplet dynamics to support membrane expansion and energy balance [4]. Conversely, dysregulated lipid metabolism—such as saturated fatty acid overload or defective lipophagy—can aggravate ER stress, creating a feed-forward loop that amplifies proteostatic and metabolic imbalance [5]. This review examines the reciprocal regulation between ER stress and lipid metabolism from the perspective of proteostasis-driven tumor adaptation and summarizes therapeutic strategies that target this adaptive network to counter cancer progression and therapy resistance.

## ER stress–driven proteostasis network in cancer cells

**Fig. 1:**
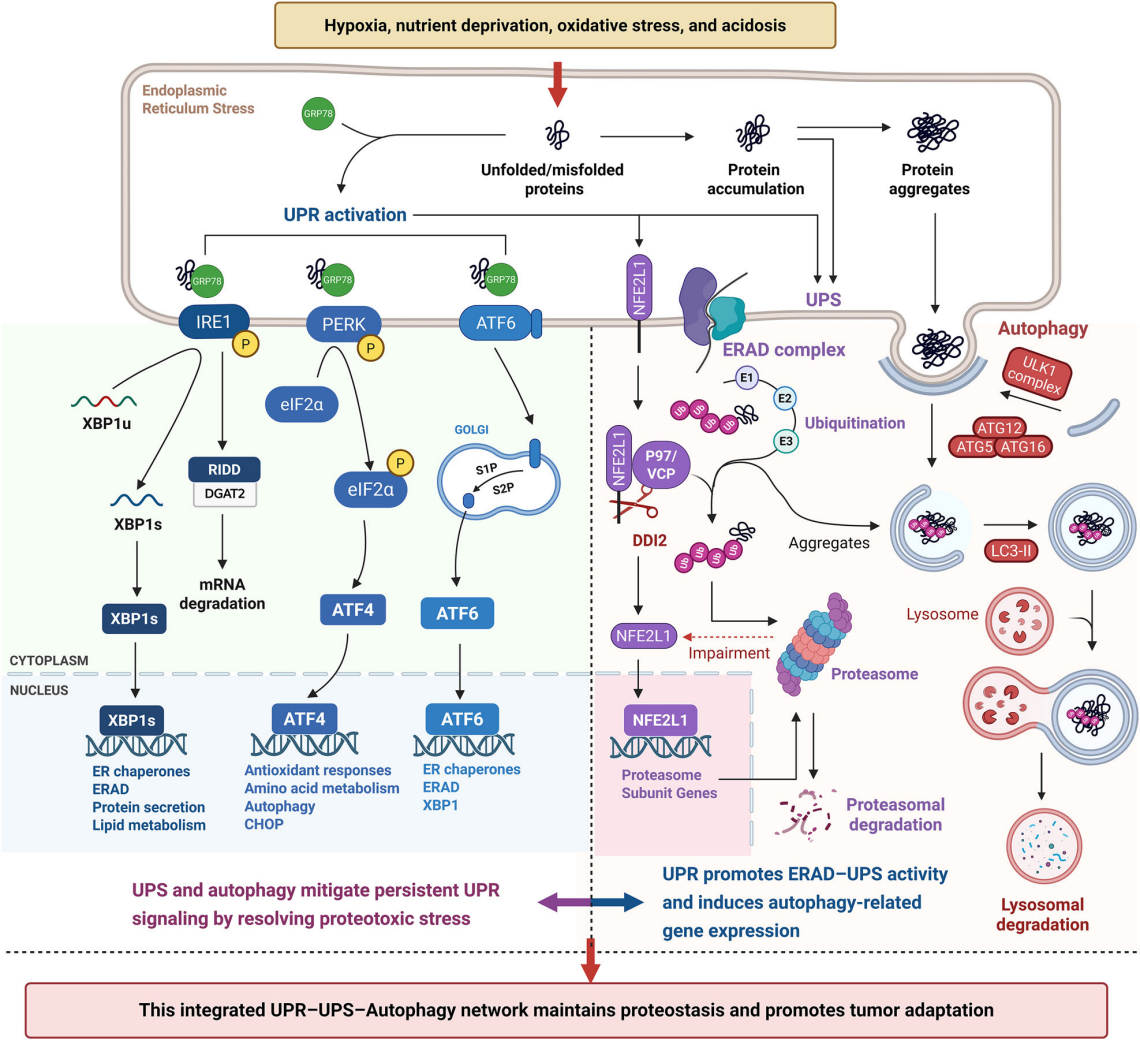
Integrated regulatory network of UPR, UPS, and autophagy in proteostasis maintenance and tumor adaptation. Tumor microenvironmental stressors such as hypoxia, nutrient deprivation, oxidative stress, and acidosis disrupt endoplasmic reticulum protein folding, resulting in the accumulation of misfolded proteins and subsequent activation of the UPR. The UPR is initiated by three ER-resident sensors—IRE1, PERK, and ATF6—whose activity is repressed under basal conditions through association with the chaperone GRP78. Upon ER stress, GRP78 dissociates to bind misfolded proteins, enabling sensor activation. Activated IRE1 catalyzes XBP1 mRNA splicing and promotes RIDD, PERK phosphorylates eIF2α to trigger ATF4-mediated transcription, and ATF6 translocates to the Golgi for proteolytic cleavage into its active form. These effectors enhance expression of ER chaperones, redox enzymes, autophagy genes, and ERAD components. Under proteotoxic stress or proteasome inhibition, the ER-anchored transcription factor NFE2L1 (NRF1) is transiently activated to upregulate proteasome subunit genes, thereby sustaining UPS capacity during ER stress. Concurrently, the UPR coordinates degradation of misfolded or aggregated proteins via the UPS and autophagy. The UPS eliminates soluble misfolded proteins through proteasomal degradation, while autophagy removes insoluble aggregates and damaged organelles via lysosomal degradation. By promoting both proteolysis and adaptive gene expression, this integrated UPR–UPS–autophagy axis alleviates proteotoxic stress, resolves persistent UPR signaling, and maintains proteostasis—ultimately enabling tumor cells to survive in hostile microenvironments and resist therapeutic pressure.

During tumor progression, malignant cells face hostile microenvironmental conditions such as hypoxia, nutrient deprivation, oxidative stress, and acidosis. These stressors impair protein folding, calcium homeostasis, and ER membrane integrity [6], triggering ER stress and activating a coordinated proteostasis network comprising the UPR, UPS, and autophagy **(**Fig. 1**)**. This adaptive machinery sustains protein homeostasis and confers metabolic plasticity, enabling cancer cells to endure persistent ER stress and resist therapy-induced challenges [7].

### UPR signaling as the initiator of ER stress–driven proteostasis

The UPR is a sensor-mediated program that detects misfolded proteins and restores ER homeostasis. It is initiated by three ER-resident sensors: inositol-requiring enzyme 1 (IRE1), protein kinase R-like ER kinase (PERK), and activating transcription factor 6 (ATF6) [8]. Under basal conditions, these sensors are maintained in an inactive state through association with the chaperone GRP78 (BiP). Upon ER stress, GRP78 dissociates to bind accumulating misfolded proteins, thereby permitting sensor activation **(**Fig. 1**)** [8].

PERK phosphorylates eIF2α, resulting in global translational attenuation to alleviate protein overload, while selectively permitting the translation of ATF4. ATF4 drives transcriptional programs involved in redox balance, amino acid metabolism, and autophagy [9]. In cancer cells, this pathway facilitates adaptation to hypoxic and nutrient-limited environments [10]. Under sustained stress, ATF4 induces the expression of C/EBP homologous protein (CHOP), a pro-apoptotic transcription factor and key marker of terminal ER stress when adaptive mechanisms are exhausted [11].

IRE1α, once activated, catalyzes the unconventional splicing of XBP1 mRNA, producing the active transcription factor XBP1s. XBP1s upregulates the expression of molecular chaperones, ER-associated degradation (ERAD) components, and lipid biosynthetic enzymes [12]. Aberrant activation of XBP1s has been reported in various cancers, where it enhances cellular adaptation to ER stress and supports tumor survival and progression [13]. In parallel, IRE1α also mediates IRE1-dependent decay (RIDD), selectively degrading mRNAs such as DGAT2, thereby modulating protein burden and metabolic output [14].

Following ER stress, ATF6 translocates to the Golgi apparatus, where it undergoes site-specific proteolysis. The cleaved cytosolic fragment migrates to the nucleus and activates the transcription of chaperones and ERAD-related genes [15]. Although less extensively characterized, ATF6 has been implicated in hypoxic tumor contexts and may also intersect with autophagy pathways to contribute to cellular adaptation [16].

### Integration of degradation and recycling pathways

Beyond proper protein folding, cellular proteostasis critically depends on two major degradative pathways: the UPS and autophagy **(**Fig. 1**)** [17]. Under ER stress, the UPR not only enhances the protein-folding capacity but also transcriptionally and functionally activates both degradation systems to clear misfolded proteins and recycle intracellular components [18].

UPR activation induces the ERAD pathway, which recognizes misfolded proteins within the ER lumen via GRP78 and facilitates their retrotranslocation into the cytosol [19]. These substrates are subsequently polyubiquitinated through the E1–E2–E3 enzymatic cascade and extracted by the p97/VCP complex for degradation by the 26S proteasome [20]. Beyond its involvement in ERAD, the UPS serves as the primary selective degradation system in the cytosol and nucleus. In cancer, the UPS is frequently reprogrammed through aberrant expression or activity of E3 ligases, influencing key oncogenic processes such as cell cycle progression, apoptosis resistance, and immune evasion [21]. This remodeling enhances tumor cell adaptability under microenvironmental stress and therapeutic challenge. Under proteotoxic stress or proteasome inhibition, the ER-anchored transcription factor NFE2L1 (NRF1) is retrotranslocated by p97/VCP and activated by DDI2 [22]. The active form translocates into the nucleus to upregulate proteasome subunit (PSM) genes, initiating a compensatory “proteasome recovery” response that restores UPS function and maintains proteostasis [23].

Autophagy, particularly macroautophagy, constitutes another essential proteolytic mechanism. It involves the sequestration of damaged organelles and aggregated proteins within double-membraned autophagosomes, which then fuse with lysosomes for degradation and nutrient recycling [24]. The process is initiated by the ULK1 complex, followed by phagophore elongation via the Atg5–Atg12–Atg16 complex, and completed by LC3-II–mediated autophagosome maturation [25]. In tumors characterized by elevated metabolic stress—such as KRAS-mutant pancreatic ductal adenocarcinoma—autophagy is markedly upregulated to maintain amino acid pools, redox homeostasis, and chemoresistance [26].

Although mechanistically distinct, the UPS and autophagy are functionally complementary. The UPS primarily targets short-lived and soluble proteins, whereas autophagy degrades large protein aggregates and damaged organelles [27]. Under ER stress, the coordinated activation of both systems forms a dynamic and flexible proteolytic network that bolsters tumor cell survival under both intrinsic and extrinsic stress conditions.

### Crosstalk and remodeling of proteostasis pathways in tumors

The UPR, UPS, and autophagy are intricately interconnected at both structural and functional levels, together forming a highly responsive and precisely regulated proteostasis network **(**Fig. 1**)**. This integrated system buffers cellular stress and sustains tumor cell survival under hostile conditions. As the central sensor of ER stress, the UPR orchestrates the activities of both the UPS and autophagy through multilayered regulatory mechanisms, enabling coordinated control over protein quality and degradation processes [8, 28].

When the accumulation of misfolded proteins exceeds the folding capacity of the ER, UPR activation enhances ERAD and promotes UPS-mediated degradation of aberrant proteins [28]. If proteasomal function is compromised or polyubiquitinated aggregates accumulate beyond clearance capacity, autophagy is activated as a compensatory mechanism to preserve proteome integrity [29]. Through this hierarchical and flexible interplay, the UPR empowers tumor cells to maintain homeostasis under fluctuating stress conditions. Notably, both the UPS and autophagy also serve as feedback regulators of UPR signaling. The UPS can limit excessive UPR activation by targeting core UPR sensors—such as IRE1α—for proteasomal degradation, thereby curbing sustained pro-death signaling [30]. Likewise, autophagy mitigates ER stress by removing misfolded proteins, damaged ER subdomains, and other stress-associated components, reducing chronic UPR activation and promoting a transition from apoptotic to adaptive responses [31]. These negative feedback loops ensure that ER stress responses remain proportional and reversible.

Importantly, this proteostasis network undergoes tumor type–specific remodeling to meet distinct metabolic and survival demands. For example, multiple myeloma cells exhibit high dependence on the UPR–UPS axis to manage excessive immunoglobulin production [32, 33]. In melanoma, ER stress–induced autophagy contributes to drug resistance, and GRP78 inhibition disrupts this adaptive mechanism, restoring chemosensitivity. Furthermore, targeting TRIM24—a regulatory node that bridges the UPS and autophagy—has been shown to restore proteasomal activity and induce apoptosis in tumor cells [34]. In summary, the synergistic integration of the UPR, UPS, and autophagy underlies the proteostatic plasticity of cancer cells. By buffering ER stress and coordinating metabolic adaptation with cell fate decisions, this adaptive network supports tumor progression and confers resistance to therapy.

## Lipid metabolic reprogramming in tumors

**Fig. 2:**
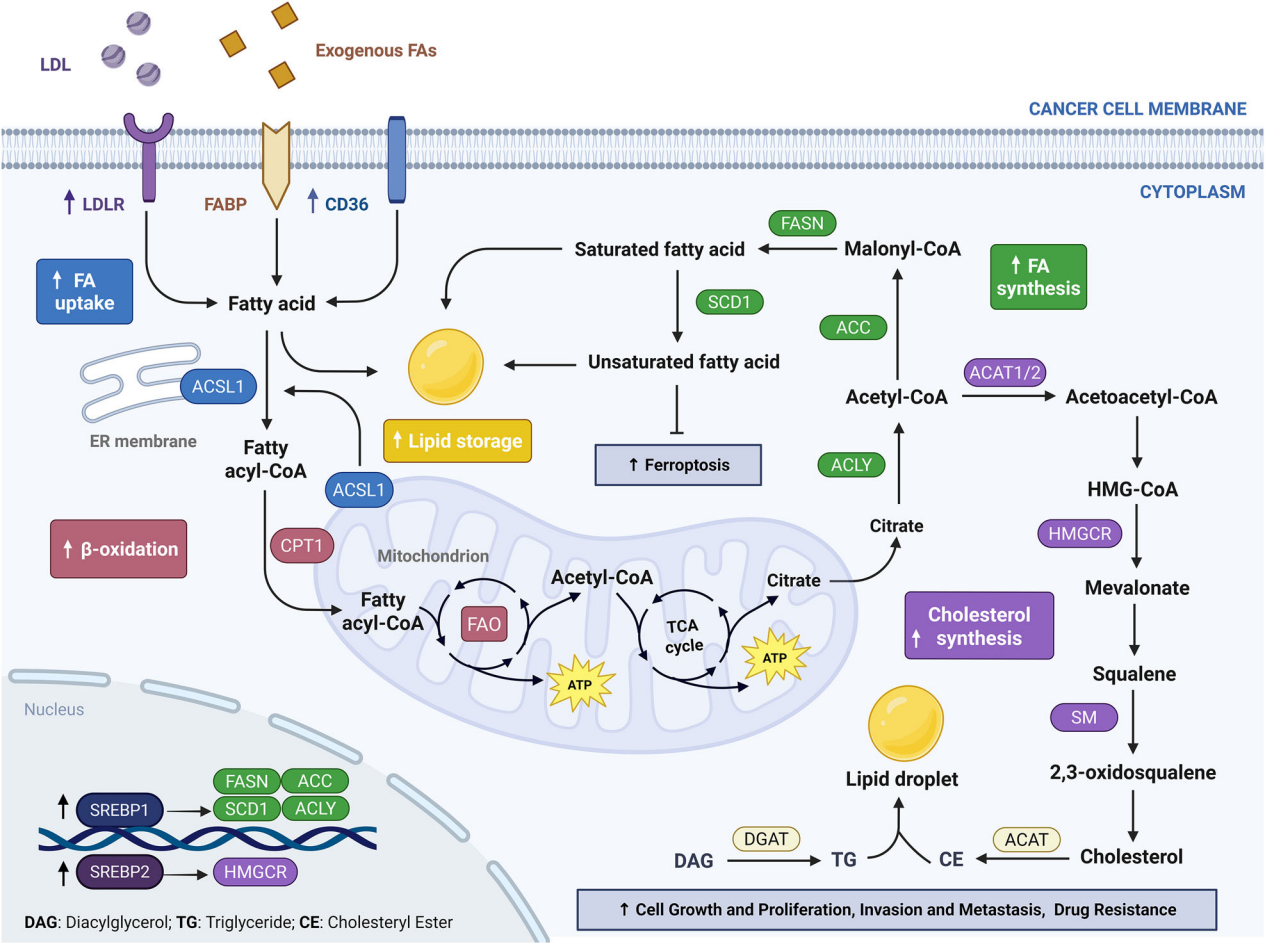
Integrated network of lipid metabolic reprogramming in cancer cells. Tumor cells undergo lipid metabolic reprogramming to sustain growth and adapt to microenvironmental stress. Fatty acid uptake is enhanced via LDLR, FABP, and CD36, while ACSL1, located at both the ER membrane and outer mitochondrial membrane (OMM), activates fatty acids for subsequent biosynthesis or oxidation. Fatty acid synthesis involves ACC, FASN, and SCD1, generating saturated and unsaturated fatty acids under SREBP1 control. Excess lipids are stored as triglycerides and cholesteryl esters in lipid droplets, which buffer oxidative stress, limit ferroptosis, and contribute to chemoresistance. Fatty acyl-CoA fuels mitochondrial β-oxidation via CPT1, supporting ATP production under nutrient stress. Acetyl-CoA also enters the mevalonate pathway, where HMGCR promotes cholesterol biosynthesis under SREBP2 regulation. Together, these pathways enhance metabolic flexibility and support tumor progression.

Lipid metabolism is essential for cellular energy storage, membrane integrity, and signaling. In cancer, it is reprogrammed to meet the demands of rapid proliferation and microenvironmental stress [35]. Tumor cells upregulate lipid synthesis, uptake, and catabolism **(**Fig. 2**)**, supporting biosynthesis, redox balance, and therapy resistance.

### Enhanced lipid synthesis and uptake in tumor cells

Aberrant activation of de novo fatty acid synthesis is a hallmark of tumor metabolic reprogramming. Rapidly proliferating cancer cells require a continuous supply of membrane phospholipids and signaling lipids, which is largely met by the upregulation of key lipogenic enzymes such as fatty acid synthase (FASN) and acetyl-CoA carboxylase (ACC) [35]. Sterol regulatory element-binding protein 1 (SREBP1), a master transcriptional regulator of lipid biosynthesis, is frequently hyperactivated in diverse malignancies, promoting the expression of FASN and ACC to support phospholipid and triglyceride synthesis, thereby facilitating membrane biogenesis and tumor cell proliferation [36, 37]. In addition, SREBP1-driven expression of stearoyl-CoA desaturase 1 (SCD1) enables the conversion of saturated fatty acids into monounsaturated fatty acids (MUFAs), which maintains membrane fluidity, alleviates lipotoxicity, and confers resistance to ferroptosis through activation of the SLC7A11/GPX4 pathway [38]. Experimental studies have shown that pharmacological inhibition or genetic silencing of FASN, SCD1, or CD36 can impair lipid metabolic flux, disrupt membrane architecture, and suppress tumor growth and chemoresistance [39].

Beyond endogenous synthesis, tumor cells also actively import exogenous fatty acids to meet metabolic demands. The fatty acid translocase CD36, which is frequently upregulated in various cancers, facilitates the uptake of long-chain fatty acids from the tumor microenvironment [40]. These imported lipids fuel β-oxidation, membrane lipid assembly, and intracellular signaling pathways that enhance tumor cell survival, invasion, and metastatic dissemination [40]. For example, in breast cancer, CD36 mediates lipid transfer from adjacent adipocytes to promote epithelial–mesenchymal transition [41], while in gastric cancer, it drives peritoneal spread via the PI3K–AKT–mTOR axis [42]. Collectively, cancer cells employ a coordinated strategy that integrates enhanced lipid synthesis, exogenous lipid uptake, and metabolic remodeling to support malignant progression and therapeutic resistance.

### Dependence on fatty acid oxidation for energy production

Fatty acid β-oxidation (FAO) is a mitochondrial process that catabolizes long-chain fatty acids to generate ATP and NADPH, thereby sustaining cellular energy homeostasis and redox balance [43]. Many tumors undergo metabolic reprogramming to enhance FAO as a compensatory energy source, particularly under nutrient limitation or within specific cancer subtypes [44]. Notably, in acidic tumor microenvironments where glycolysis is suppressed, cancer cells increase FAO activity to maintain energy production and viability [45]. Carnitine palmitoyltransferase 1 A (CPT1A), the rate-limiting enzyme responsible for mitochondrial import of long-chain fatty acids [46], is frequently upregulated in tumors and serves as a key driver of FAO activation (Fig. 2). In nasopharyngeal carcinoma, high CPT1A expression promotes cell proliferation and anchorage-independent growth [47].

Critically, enhanced FAO has been closely linked to therapeutic resistance. In high-grade serous ovarian cancer, cisplatin-resistant cells exhibit elevated fatty acid uptake and CPT1A-mediated FAO, while CPT1A inhibition effectively restores chemosensitivity [48]. Similar observations have been reported in radiotherapy-resistant breast and nasopharyngeal cancers, where FAO blockade resensitizes tumor cells to radiation-induced apoptosis [49]. FAO also contributes to immune evasion. In melanoma, Wnt5a–PPARγ signaling promotes CPT1A expression and FAO activation, which in turn impairs cytotoxic T cell function. Targeting this axis enhances the efficacy of anti–PD-1 immunotherapy [50]. Together, FAO endows tumors with metabolic flexibility and stress tolerance, supporting continued growth under unfavorable conditions and contributing to resistance against diverse anticancer therapies.

### Dysregulated cholesterol homeostasis

Cholesterol is an essential structural component of cellular membranes, where it modulates membrane fluidity, maintains lipid raft integrity, and serves as a precursor for steroid hormones and other signaling molecules [51]. In cancer, cholesterol metabolism is frequently reprogrammed to meet the demands of rapid proliferation and oncogenic signaling **(**Fig. 2**)**.

The transcription factor SREBP2 is commonly upregulated in tumors and activates key enzymes of the mevalonate pathway, including HMG-CoA reductase (HMGCR), to drive de novo cholesterol biosynthesis. Concurrently, expression of the LDL receptor (LDLR) is often elevated to facilitate the uptake of exogenous cholesterol [52]. Under cholesterol-deprived conditions, the SREBP2–SCAP complex translocates to the Golgi apparatus, where SREBP2 undergoes proteolytic cleavage to release its active fragment, which in turn induces the expression of genes involved in cholesterol metabolism [53].

Accumulation of intracellular cholesterol enhances membrane stability and promotes lipid raft formation, thereby facilitating the clustering and activation of oncogenic signaling pathways such as Wnt–β-catenin [54]. To mitigate lipid-induced toxicity, cancer cells often esterify excess cholesterol into neutral lipid droplets, which sequester oxidizable lipids and suppress ferroptosis [51]. This buffering capacity not only protects against oxidative stress but also supports metastatic potential. Indeed, studies have demonstrated that elevated intracellular cholesterol levels significantly inhibit ferroptotic cell death and enhance tumorigenic capacity [55]. In summary, reprogrammed cholesterol metabolism contributes to tumor growth, signal transduction, and stress tolerance. Its central role in both structural and signaling networks makes cholesterol homeostasis a compelling target for therapeutic intervention in cancer.

### Lipid droplet dynamics and stress adaptation

Lipid droplets (LDs) are neutral lipid storage organelles that accumulate extensively in tumor cells, reflecting enhanced lipid synthesis and uptake [56]. By sequestering excess fatty acids and cholesterol in the form of triglycerides and cholesteryl esters, LDs serve dual functions: acting as energy reservoirs and buffering against lipotoxicity. The mobilization of LD-stored lipids provides substrates for β-oxidation and membrane biosynthesis, thereby sustaining tumor growth under metabolic stress [56].

LDs also interact with the endoplasmic reticulum and mitochondria to coordinate lipid trafficking and redistribution, optimizing resource utilization during stress adaptation [57]. Importantly, LDs protect cells from oxidative damage by trapping polyunsaturated fatty acids (PUFAs), which helps limit lipid peroxidation and ferroptotic cell death [58]. In hypoxic prostate cancer cells, increased LD formation combined with reduced PUFA incorporation into membrane phospholipids contributes to ferroptosis resistance [59].

In addition to their metabolic functions, LDs may contribute to chemoresistance by sequestering hydrophobic chemotherapeutic agents, thereby reducing their intracellular availability. This mechanism is supported by evidence from doxorubicin-resistant MCF-7 breast cancer cells, which exhibit elevated LD accumulation [60]. Collectively, LDs function as adaptive metabolic buffers that enable tumor cells to withstand nutrient deprivation, oxidative stress, and therapeutic pressure, underscoring their critical role in metabolic flexibility and cancer progression.

## Bidirectional coupling between ER stress and lipid metabolism

**Fig. 3:**
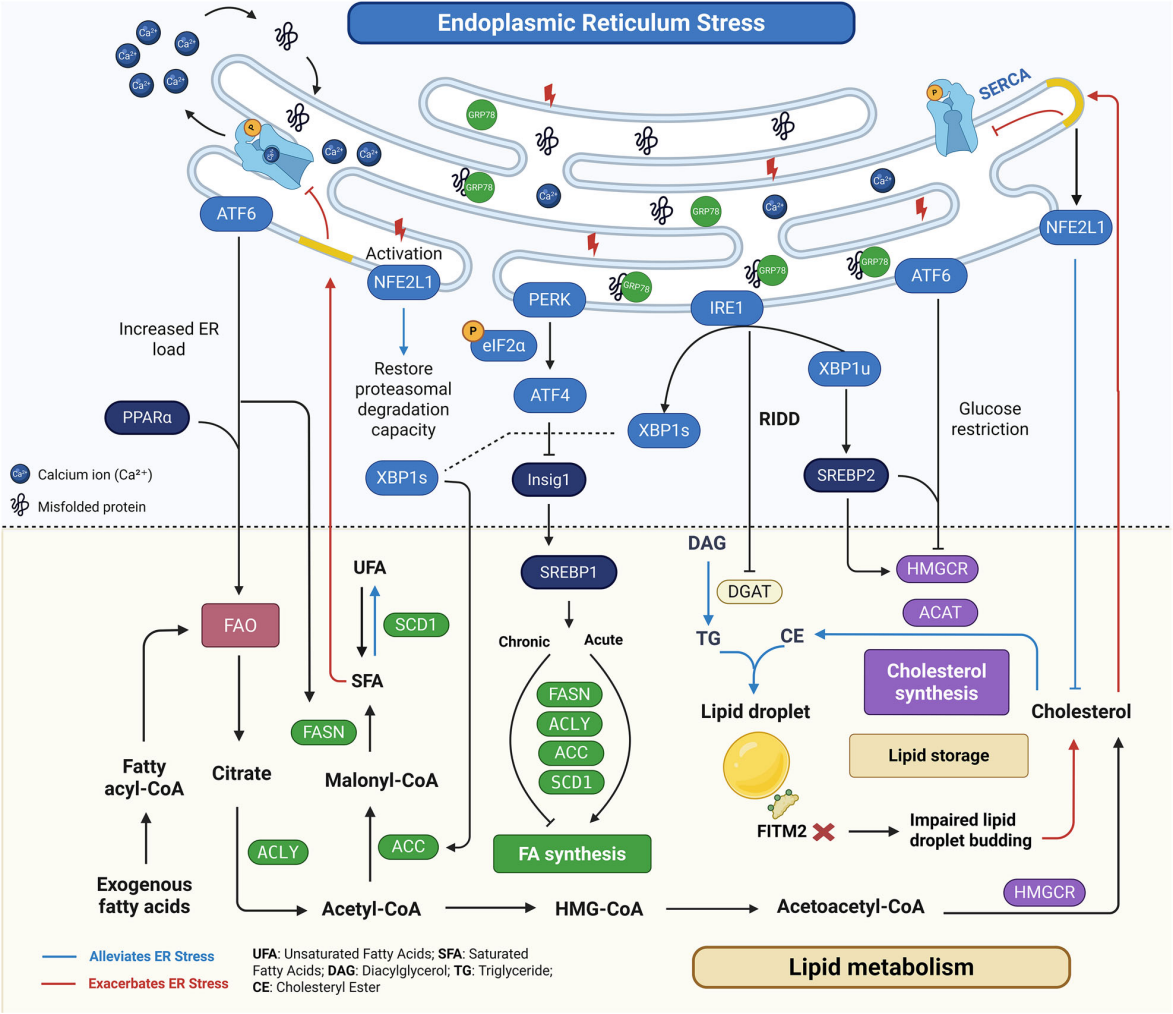
Bidirectional crosstalk between endoplasmic reticulum stress and lipid metabolism in cancer cells. This figure depicts the reciprocal regulation between ER stress and lipid metabolism in tumors. UPR signaling promotes lipid biosynthesis and remodeling via PERK–ATF4–SREBP1, IRE1–XBP1s, and ATF6-mediated pathways. These include upregulation of fatty acid and cholesterol synthesis, lipid droplet formation, and adaptation to metabolic stress. In parallel, chronic ER stress activates the ER-anchored transcription factor NFE2L1, which restores proteasome function and senses cholesterol overload within the ER membrane to maintain proteostasis and lipid homeostasis. Conversely, lipid imbalance—such as saturated fatty acid overload or cholesterol accumulation—disrupts ER membrane properties and calcium homeostasis, triggering or exacerbating ER stress. Lipid droplets buffer lipotoxicity and mitigate oxidative stress, serving as protective compartments. This bidirectional loop supports tumor survival, metabolic plasticity, and therapeutic resistance.

In tumor cells, the ER functions as a central hub integrating protein folding with lipid metabolism [61]. ER stress and lipid metabolism engage in a reciprocal feedback loop, where the UPR reprograms lipid pathways and lipid imbalance amplifies stress signaling to enhance tumor adaptability **(**Fig. 3**)**.

### ER stress–mediated lipid metabolic reprogramming in cancer

The PERK–eIF2α–ATF4 pathway plays a central role in regulating lipid biosynthesis. By suppressing the translation and stability of Insig‑1, it relieves inhibition on SREBP1, thereby promoting the transcription of lipogenic genes such as ACC and SCD1 [62]. Loss of ATF4 markedly impairs triglyceride synthesis and downregulates key lipogenic enzymes, resulting in defective lipid storage, accumulation of free fatty acids, and exacerbation of oxidative and ER stress [63]. Under prolonged or severe ER stress, this pathway shifts toward global suppression of both protein and lipid synthesis to alleviate ER burden, reflecting its temporally dynamic and bidirectional regulatory nature [64, 65].

Although FAO is oxygen-dependent and thus impaired under severe hypoxia, it can be sustained to a certain extent under specific microenvironmental contexts (e.g., perivascular or intermittent hypoxia) through adaptive UPR signaling [66, 67]. The PERK–ATF4 axis contributes to this compensatory adaptation by enhancing antioxidant responses, autophagy, and mitochondrial remodeling, thereby supporting survival of cancer stem-like or drug-resistant populations [68].

The IRE1α–XBP1 axis regulates lipid homeostasis through two complementary mechanisms: transcriptional activation of lipogenesis via XBP1s, and lipid droplet modulation via RIDD. In tumor cells, XBP1s directly induces ACC expression, thereby promoting fatty acid synthesis [12]. Additionally, unspliced X-box binding protein 1 (XBP1u) stabilizes SREBP2 and upregulates HMGCR, thereby promoting cholesterol biosynthesis and membrane remodeling [69]. In triple-negative breast cancer, hyperactivation of this pathway enables metabolic adaptation under nutrient deprivation and harsh microenvironmental conditions [14]. Concurrently, RIDD-mediated degradation of mRNAs such as DGAT2 limits lipid droplet accumulation and prevents lipotoxicity [14, 70]. Notably, in IRE1α-deficient cells, further suppression of DGAT2 partially restores proliferation, highlighting the critical role of the RIDD–DGAT2 axis in maintaining lipid balance and adaptive survival [71].

ATF6 also contributes to lipid metabolic regulation [72, 73]. Under nutrient-restricted conditions, such as glucose deprivation, its cleaved and active form (ATF6f) forms a complex with SREBP2 to suppress cholesterol biosynthesis [72]. In contrast, under nutrient surplus or elevated ER load, ATF6 promotes the expression of lipogenic genes including FASN and cooperates with PPARα to enhance fatty acid β-oxidation, thereby reducing triglyceride accumulation and sustaining lipid homeostasis [73]. In colorectal cancer, elevated ATF6 activity has been observed in tumor tissues, where it reprograms fatty acid synthesis in intestinal epithelial cells [74], underscoring its role in tumor-associated lipid metabolic remodeling. In summary, these pathways establish a lipid–stress regulatory network that maintains membrane integrity, energy balance, and tumor adaptation **(**Fig. 3**)**.

### Lipid metabolic imbalance exacerbates ER stress in cancer

In many cancers, reprogrammed lipid metabolism leads to excessive uptake and synthesis of fatty acids, particularly saturated fatty acids (SFAs), which potently induce ER stress and UPR activation [75]. SFAs such as palmitic acid incorporate into ER membranes, increasing rigidity and reducing fluidity. They also disrupt calcium homeostasis by inhibiting SERCA pumps or promoting calcium efflux, resulting in ER calcium depletion, chaperone inactivation, and misfolded protein accumulation [76]. In ovarian cancer, SCD1 inhibition leads to SFA accumulation and robust ER stress–mediated apoptosis, which can be rescued by oleic acid supplementation [77]—highlighting the critical role of fatty acid desaturation in preserving ER function.

Abnormal cholesterol accumulation is another key contributor to ER stress. Under physiological conditions, ER membranes maintain extremely low cholesterol levels, rendering them sensitive to fluctuations [52]. In cancer, enhanced cholesterol uptake and de novo synthesis support membrane biogenesis but also raise the risk of cholesterol overload [78]. Excess unesterified cholesterol, if not efficiently sequestered into lipid droplets via ACAT, integrates into the ER membrane and directly activates transmembrane UPR sensors such as IRE1α and PERK [79], triggering “cholesterol stress” [80]. In hepatocellular carcinoma and other cholesterol-addicted tumors, sustained ER cholesterol accumulation drives chronic ER stress and promotes tumor progression [81].

Disrupted LD dynamics also exacerbate ER stress. As ER-derived organelles, LDs buffer excess neutral lipids and protect against lipotoxicity [82]. Loss of FITM2, a key regulator of LD budding, impairs LD biogenesis and causes the retention of triglycerides and cholesterol esters within the ER, which triggers UPR activation and morphological abnormalities [83]. Restoration of FITM2 expression rescues this phenotype, indicating a direct link between defective LD formation and ER stress. Furthermore, limited LD mobilization can impair ER–organelle contact sites, altering membrane tension and disrupting intracellular signaling [84]. Although the UPR can trigger compensatory mechanisms—such as upregulation of DGAT enzymes [85] or activation of lipophagy [86]—persistent imbalance often overrides these responses.

In summary, dysregulated lipid metabolism aggravates ER stress in tumor cells through several converging mechanisms: SFA accumulation alters membrane architecture and calcium handling; cholesterol overload directly activates UPR sensors; and impaired LD biogenesis or turnover limits lipid buffering capacity **(**Fig. 3**)**. Although UPR-driven compensatory pathways attempt to restore balance, chronic lipid metabolic stress frequently overwhelms these systems, resulting in a maladaptive “lipotoxicity–ER stress” feedback loop. This vicious cycle enhances tumor adaptability, fosters therapeutic resistance, and promotes malignant progression.

### Bidirectional coupling between ER stress and lipid metabolism in cancer

ER stress and lipid metabolism are tightly coupled through a bidirectional feedback loop that shapes the adaptive capacity of tumor cells **(**Fig. 3**)**. On one hand, activation of the UPR reprograms lipid metabolism to mitigate ER burden and restore homeostasis. On the other, dysregulated lipid metabolism modulates the strength and persistence of UPR signaling [8]. This dynamic interaction enables cancer cells to adapt to metabolic stress, yet under chronic conditions, this adaptive loop may transform into a maladaptive cycle that promotes tumor progression.

Under acute ER stress, the UPR induces fatty acid and triglyceride synthesis to expand the ER membrane and dilute unfolded protein concentrations, thereby promoting transient cytoprotection [75]. However, sustained stress or excessive lipid biosynthesis results in the accumulation of saturated fatty acids, cholesterol, and lipid droplets—factors that exacerbate ER membrane dysfunction and perpetuate UPR activation [4]. This feedback enhances metabolic plasticity. For instance, under nutrient deprivation or hypoxia, UPR signaling shifts toward fatty acid oxidation to maintain energy production. Conversely, when membrane synthesis is prioritized, UPR promotes lipid anabolism and ER biogenesis. Such metabolic flexibility enables tumor cells to rapidly switch between anabolic and catabolic lipid programs in response to fluctuating environmental cues, facilitating sustained growth and survival [87]. Beyond the classical UPR pathways, chronic ER stress can also induce the processing and activation of NFE2L1 [22]. Activated NFE2L1 restores proteasomal degradation to maintain proteostasis and regulates ER-membrane cholesterol under overload, preventing lipotoxicity and preserving ER integrity [88, 89]. Thus, NFE2L1 acts as a critical regulator that integrates the UPS and lipid metabolic transcriptional networks, sustaining ER homeostasis and conferring survival advantages to tumor cells under metabolic stress and therapeutic pressure.

Importantly, this ER stress–lipid metabolism coupling also plays a pivotal role in mediating therapeutic resistance. Persistent UPR activation elevates chaperones and anti-apoptotic pathways that buffer drug-induced stress. Concurrently, UPR-driven programs mediated by XBP1s and SREBP1 induce FASN and ACC, enhancing saturated fatty-acid and cholesterol synthesis [90]. These lipid changes alter membrane fluidity and integrity, modulating drug transport, and decreasing intracellular drug accumulation. Furthermore, elevated lipid pools provide substrates for autophagosome and lysosome membranes, reinforcing autophagy-mediated damage clearance. Multiple studies have reported that UPR activation and lipogenic enzyme overexpression frequently co-occur in chemoresistant tumors, forming an integrated stress-metabolism axis that fortifies cellular defenses [91, 92].

In conclusion, the bidirectional interaction between ER stress and lipid metabolism endows tumor cells with enhanced adaptability, metabolic agility, and therapeutic resistance. By integrating stress signaling with lipid metabolic remodeling, this coupling sustains tumor survival under adverse conditions and represents a promising node for therapeutic intervention.

## Therapeutic potential of ER stress-lipid metabolism mechanisms

### Small-molecule compounds

***Targeting ER stress*** GRP78 inhibitors such as HA15 [93], OSU-03012 [94] and KP1339 [95] disrupt ER homeostasis and trigger apoptosis, with KP1339 also inducing immunogenic cell death [96] and showing clinical potential in colorectal cancer [97]. IRE1α inhibitors including Sunitinib [98], STF-083010 [99], and Toyocamycin [100] have shown antitumor efficacy in various preclinical cancer models. Among them, the next-generation compound ORIN1001 has progressed to phase I clinical trials in patients with advanced solid tumors, exhibiting a favorable safety profile and promising preliminary efficacy [101]. For the PERK branch, early ATP-competitive inhibitors such as GSK2606414 displayed strong antitumor effects but were hindered by systemic toxicity [102, 103]. In contrast, second-generation inhibitors like GSK2656157 have shown improved safety and efficacy, suppressing tumor growth in models of hepatocellular carcinoma [104], breast cancer [105], and pancreatic neuroendocrine tumors [106]. The advanced PERK inhibitor HC-5404 has entered phase I/II trials for advanced solid tumors, further underscoring the translational value of PERK pathway targeting [64]. The ATF6 branch has also been selectively targeted. Ceapin-A7, a selective ATF6 inhibitor, has been shown to induce apoptosis and sensitize diffuse large B-cell lymphoma cells to Adriamycin treatment [107]. Together, these compounds exemplify the therapeutic potential of targeting various branches of the ER stress response. A summary of key small-molecule modulators involved in the ER stress–lipid metabolism axis is provided in Table 1.

**Table 1:** Representative Therapeutic Strategies Targeting the ER stress–Lipid Metabolism Axis in Cancer.

a						
Category	Agent	Target/Pathway	Mechanism	Cancer Type	Status	Ref.
Small molecule	HA15	GRP78 / UPR–Autophagy	Inhibits GRP78, triggers ER stress and autophagy, leading to apoptosis	Lung cancer	Preclinical (in vitro / in vivo)	[93]
	OSU-03012	GRP78/BiP	Blocks GRP78 activity and expression, inducing apoptosis	Glioblastoma	Preclinical (in vitro)	[94]
	KP1339 / IT‑139 (BOLD‑100)	GRP78 / PERK–eIF2α–ICD, caspase-8	Inhibits GRP78, disrupts ER homeostasis, activates caspase-8 apoptosis	Colorectal cancer, multiple cancer cell lines	Preclinical (in vitro) Phase I clinical trial (NCT01415297)	[95–97]
	Sunitinib	IRE1α kinase	Blocks IRE1α activity, synergizes with chemotherapy	Pancreatic cancer, multiple myeloma	FDA-approved for other indications; Preclinical (in vitro/in vivo)	[98]
	STF‑083010	IRE1α–XBP1–CHOP–Bim	Inhibits XBP1 splicing, upregulates CHOP and Bim, activates apoptosis	Ovarian cancer	Preclinical (in vitro)	[99]
	Toyocamycin	IRE1α–UPR–VEGF axis	Inhibits IRE1α RNase, suppresses VEGF induction, blocks hypoxic growth	Renal cell carcinoma	Preclinical (in vitro)	[100]
	ORIN1001	IRE1α–XBP1 RNase pathway	Inhibits IRE1α RNase, blocks XBP1, boosts therapy sensitivity	Advanced solid tumors	Phase I clinical trials (NCT05154201); PopPK model established in Chinese patients	[101]
	GSK2606414	PERK / UPR pathway	Inhibits PERK, blocks UPR, enhances apoptosis sensitivity	Multiple Myeloma	Preclinical (in vitro)	[102, 103]
	GSK2656157	PERK–eIF2α–CHOP	Inhibits PERK–eIF2α–CHOP to regulate ER stress–related apoptosis or immune exhaustion	Hepatocellular carcinoma, breast cancer, pancreatic neuroendocrine tumors	Preclinical (in vitro / in vivo)	[104–106]
	HC‑5404	PERK kinase	Inhibits PERK, prevents VEGFR-TKI stress adaptation, enhances antitumor effects	Renal cell carcinoma, advanced solid tumors	Preclinical (in vivo); Phase I clinical trials (NCT04834778)	[62]
	Ceapin-A7	ATF6	Blocks ATF6 Golgi translocation and cleavage, induces stress and apoptosis	Diffuse large B-cell lymphoma	Preclinical (in vitro)	[107]
	SSI-4	SCD1 / ER stress axis	Induces lipid imbalance–driven ER stress and ferroptosis	Acute myeloid leukemia	Preclinical (in vitro */* in vivo)	[108]
	TVB-2640	FASN	Inhibits FASN, disrupts lipid metabolism	KRAS-mutant NSCLC, breast, and ovarian cancer	Phase I/II clinical trials (NCT02223247, NCT03808558)	[109]
	Statins	HMGCR / RAS prenylation / ER stress	Block RAS prenylation, induce ER stress and ICD, activate CD8⁺ T cells	KRAS-mutant tumors	Preclinical (in vitro)	[111]

b						
Category	Agent (chemical class)	Target/Pathway	Mechanism	Cancer Type	Status	Ref.
Natural product	Celastrol (terpenoid)	ER stress (PERK/CHOP), intrinsic apoptosis	Induces ER stress and UPR, triggers cell cycle arrest and apoptosis	Hepatocellular carcinoma	Preclinical (in vitro / in vivo)	[112]
	Resveratrol (polyphenols)	FASN, SREBP1, HMGCR, SIRT1–AMPK, PI3K/AKT/mTOR, sphingolipid metabolism	Suppresses lipid/cholesterol synthesis, activates SIRT1–AMPK, modulates sphingolipid balance and PI3K–AKT–mTOR signaling	Breast, colon, liver, gastric, pancreatic, glioblastoma, ovarian cancers	Preclinical (in vitro / in vivo)	[113]
	β-elemene (terpenoid)	ER stress; DR5/Caspase-8/FADD (DISC, lipid rafts)	Induces ER stress–related apoptosis, disrupts lipid rafts, enhances TRAIL sensitivity	NSCLC, Gastric cancer	Preclinical (in vitro */* in vivo)	[114, 115]
	Isobavachalcone (flavonoid)	TrxR1 / ROS / ER Stress	Inhibits TrxR1, elevates ROS, induces ER stress and apoptosis	Prostate cancer	Preclinical (in vitro)	[116]
	Myricetin (flavonoid)	ER Stress (GRP78/CHOP), DNA Damage (γ-H2AX)	Triggers ER stress and DNA damage, activates apoptosis	Ovarian cancer	Preclinical (in vitro)	[117]
	Baicalein (flavonoid)	ER Stress (CHOP/eIF2α/IRE1α), Bcl-2, Autophagy	Activates CHOP; suppresses Bcl-2, induces ER stress–mediated apoptosis and autophagy	Hepatocellular carcinoma	Preclinical (in vitro)	[118]
	Astragalus flavonoids (flavonoid)	ER Stress (IRE1/XBP1/CHOP), IL-17, RORγt, Immune indices	Inhibit IRE1–XBP1, increase CHOP, modulate immune markers, suppress tumor	Lewis lung cancer	Preclinical (in vivo)	[119]

c						
Category	Agent	Target/Pathway	Mechanism	Cancer Type	Status	Ref.
Natural product	Onion flavonoids (flavonoid)	Lipid metabolism (apoB, TC)	Suppress tumor growth and reduce blood lipids	Colorectal cancer	Preclinical (in vivo)	[120]
	Berberine (alkaloid)	SCAP/SREBP-1, Wnt/β-catenin, FABPs, PPARα, fatty acid metabolism	Inhibits SCAP/SREBP1 and FABPs/PPARα, suppresses lipogenesis and induces apoptosis	Colon and gastric cancer	Preclinical (in vitro / in vivo)	[121, 122]
	Cordycepin (Cordyceps)	Lipid metabolism (SREBF1, FASN, ACC1), EMT, AMPK/MAPK signaling	Activates AMPK/MAPK, inhibits lipogenesis and EMT, induces apoptosis	Gastric cancer	Preclinical (in vitro)	[123]
Combination Strategies	4μ8C	IRE1α, ER stress, Lipid metabolism	Blocks ER stress and lipid metabolism, promotes metabolic exhaustion and enhances doxorubicin toxicity	Hepatocellular carcinoma	Preclinical (in vitro / in vivo)	[124]
	Resveratrol (polyphenols)	ER stress (PERK/eIF2α/ATF4/CHOP), apoptosis, cell cycle arrest	Synergizes with cisplatin to inhibit cell viability, induce ER stress–mediated apoptosis	Gastric adenocarcinoma	Preclinical (in vitro)	[125]
	A939572	SCD1, ER stress, CD8 + T cells, immune microenvironment	SCD1 inhibition reduces ER stress; PD-1 blockade activates T cells, improves immune response	Colon cancer, fibrosarcoma, breast cancer	Preclinical (in vivo)	[126]
	Orlistat	FASN, ER stress, Cancer stemness	Inhibits fatty acid synthesis, induces ER stress and apoptosis, reduces stemness, improves gemcitabine response	Pancreatic cancer	Preclinical (in vitro / in vivo)	[127]

***Targeting Lipid Metabolism*** Lipid metabolism–targeting agents also exhibit considerable therapeutic potential in oncology. Inhibition of SCD1 selectively compromises tumor cell viability while sparing normal cells, underscoring its value as a promising metabolic target [77]. In acute myeloid leukemia, the clinical-grade SCD1 inhibitor SSI-4 markedly suppresses leukemic growth through lipid desaturation blockade and ER stress–associated ferroptosis [108]. Similarly, FASN inhibitors have shown translational promise. TVB-2640, currently under evaluation in multiple phase I/II clinical trials for KRAS-mutant cancers, has demonstrated favorable safety and preliminary therapeutic efficacy, supporting its use as either monotherapy or in combination regimens [109]. Furthermore, statins—clinically approved HMGCR inhibitors—have shown potential benefits in non–small cell lung cancer (NSCLC). A meta-analysis encompassing more than 51,000 NSCLC patients reported a significant improvement in overall survival among statin users [110]. Mechanistically, statins impair RAS protein prenylation in KRAS-mutant tumors, intensify ER stress, and induce immunogenic cell death (ICD), thereby enhancing CD8⁺ T-cell–mediated antitumor immunity and potentially overcoming resistance to PD-1 blockade therapies [111].

### Natural products

***Polyphenols and terpenoids*** Polyphenols and terpenoids exert anticancer effects mainly by modulating either ER stress or lipid metabolism, with a few compounds displaying dual activity. For instance, celastrol, a representative terpenoid, primarily activates the UPR and intrinsic apoptotic pathways, leading to ER stress–mediated apoptosis and tumor suppression in hepatocellular carcinoma [112]. In contrast, resveratrol, a well-studied polyphenol, mainly targets lipid metabolism by inhibiting FASN and SREBP1, activating the SIRT1–AMPK axis, and altering sphingolipid and cholesterol metabolism, thereby inducing metabolic stress and apoptosis in tumor cells [113]. Notably, β-elemene exhibits dual regulatory activity, as it induces ER stress–dependent apoptosis in NSCLC [114] and enhances TRAIL-induced apoptosis in gastric cancer by promoting lipid raft–dependent DISC assembly [115]. Collectively, these findings indicate that while most polyphenols and terpenoids act through a single pathway, certain compounds possess dual-target potential.

***Flavonoids*** Flavonoids represent a diverse class of bioactive compounds that influence cancer progression by modulating ER stress or lipid metabolism. Several flavonoids predominantly activate ER stress, such as isobavachalcone, myricetin, and baicalein, which induce ER stress–mediated apoptosis in prostate [116], ovarian [117], and hepatocellular carcinoma cells [118], respectively, via upregulation of UPR markers including GRP78, ATF4, CHOP, and XBP1. In vivo, flavonoid extracts from *Astragalus* further downregulate the IRE1α–XBP1 pathway and GRP78, while upregulating CHOP, thereby promoting ER stress–induced apoptosis [119]. Conversely, other flavonoids mainly target lipid metabolism, with onion-derived flavonoids shown to reduce serum apoB and cholesterol levels in hyperlipidemic colorectal cancer models [120].

***Alkaloids and other natural products*** Alkaloids and other structurally diverse natural products also display anticancer activity through modulation of ER stress and/or lipid metabolism. For example, berberine suppresses SCAP/SREBP1 signaling to inhibit lipogenesis and tumor proliferation in colon cancer [121], and downregulates FABPs and PPARα to induce fatty acid accumulation and apoptosis in gastric cancer [122]. In contrast, cordycepin, a nucleoside analog derived from *Cordyceps*, regulates both pathways by activating AMPK to downregulate SREBP1 and FASN, disrupting lipid rafts, and inducing ER stress–mediated apoptosis in gastric cancer cells [123]. Thus, alkaloids include compounds that primarily affect lipid metabolism, such as berberine, as well as dual-target agents like cordycepin. Representative natural products and their regulatory effects on ER stress and/or lipid metabolism are summarized in Table 1.

### Combination therapy strategies

Tumor cells exhibit high metabolic plasticity and robust stress adaptation, rendering single-target therapies often insufficient for sustained efficacy. Dual targeting of the ER stress–lipid metabolism axis can disrupt both proteostatic and metabolic homeostasis, thereby weakening tumor survival and achieving synergistic effects in chemosensitization and immunotherapy. Representative combination strategies are summarized in Table 1.

Chemotherapy sensitization represents the most direct application of this approach. The IRE1α inhibitor 4μ8C, when combined with doxorubicin in hepatocellular carcinoma, downregulates lipolytic enzymes, depletes energy stores, and amplifies ER stress–mediated apoptosis, resulting in stronger cytotoxicity than monotherapy [124]. Similarly, the natural compound resveratrol enhances the cytotoxic effect of cisplatin in AGS gastric cancer cells by inducing ER stress–mediated apoptosis and G2/M arrest [125]. Its reported ability to modulate lipid metabolism further supports its potential to act through the ER stress–metabolism axis [113].

Beyond chemotherapy, the ER stress–lipid metabolism interplay is also implicated in tumor immune evasion. Lipid dysregulation can aggravate ER stress, while sustained ER stress impairs antigen presentation and T-cell function [126]. Targeting this crosstalk can help restore antitumor immunity; for instance, the SCD1 inhibitor A939572 alleviates ER stress–induced immunosuppression, inhibits β-catenin signaling, and reactivates CD8⁺ T cells, thereby enhancing anti-PD-1 immunotherapy efficacy [126].

Moreover, several clinically approved drugs have shown translational potential. The FASN inhibitor orlistat, approved by the FDA, synergizes with gemcitabine in pancreatic cancer by inducing ER stress and reducing tumor stemness, thereby overcoming chemoresistance [127]. Collectively, dual targeting of ER stress and lipid metabolism may not only improve chemotherapy efficacy but also potentiate immunotherapy, providing a feasible and promising avenue for clinical translation.

## Summary

ER stress and lipid metabolic reprogramming are critical adaptive mechanisms that enable tumor cells to survive and persist under hostile microenvironmental conditions. In this review, we highlight the central role of the UPR in orchestrating proteostasis and lipid metabolism during stress adaptation. Specifically, we describe how the IRE1, PERK, and ATF6 branches coordinate to alleviate ER burden, sustain protein homeostasis, and remodel lipid networks. In parallel, we discuss the complementary functions of the UPS and autophagy, which cooperate with the UPR to maintain proteostatic and metabolic balance, underscoring the integrated nature of tumor adaptive regulation. Driven by oncogenic stress and uncontrolled proliferation, cancer cells exhibit persistent UPR activation and extensive lipid metabolic rewiring, including enhanced fatty acid and cholesterol biosynthesis, increased lipid uptake, and lipid droplet accumulation. These processes ensure membrane biogenesis, energy production, and redox balance. However, excessive accumulation of lipotoxic metabolites such as saturated fatty acids and free cholesterol disrupts ER membrane integrity and protein folding, exacerbating ER stress and forming a self-reinforcing loop that amplifies tumor adaptation and therapeutic resistance.

Targeting the ER stress–lipid metabolism axis has shown promising potential in preclinical cancer models. Small-molecule modulators of UPR signaling and inhibitors of lipid metabolic enzymes have demonstrated anticancer efficacy by destabilizing proteostasis and metabolic homeostasis. Additionally, natural products—including polyphenols, terpenoids, flavonoids, and alkaloids—exhibit dual regulatory effects on ER stress and lipid metabolism, representing attractive multi-targeted therapeutic candidates.

Despite these advances, significant barriers remain for clinical translation, including poor bioavailability, off-target toxicity, and tumor metabolic plasticity that limits single-agent efficacy. Emerging delivery platforms such as lipid-based nanoparticles, polymeric carriers, and antibody–drug conjugates offer promising avenues for tumor-selective and organelle-targeted delivery. Moreover, rationally designed combination therapies that concurrently target UPR pathways, lipid metabolism, and conventional treatment modalities—such as chemotherapy, radiotherapy, and immunotherapy—hold the potential to overcome resistance and suppress tumor adaptability. The development of reliable biomarkers to stratify patients based on ER stress or lipid metabolic profiles will be essential for advancing precision oncology. Future research should leverage single-cell multi-omics technologies, AI-assisted drug discovery, and well-designed clinical trials to accelerate the translation of these therapeutic strategies.

In conclusion, a deeper mechanistic understanding of ER stress–lipid metabolism crosstalk will illuminate how tumor cells adapt to metabolic and therapeutic stress. Exploiting this adaptive network as a therapeutic vulnerability holds promise for improving cancer treatment outcomes.
